# The influence of fatigue and chronic low back pain on muscle recruitment patterns following an unexpected external perturbation

**DOI:** 10.1186/s12891-017-1523-3

**Published:** 2017-04-19

**Authors:** Júlia Jubany, Lieven Danneels, Rosa Angulo-Barroso

**Affiliations:** 1 Institut Nacional d’Educació Física de Catalunya, (INEFC), University of Barcelona, Avinguda de l’Estadi 12-22, Anella Olímpica, 08038 Barcelona, Spain; 2 Manresa University (Universitat de Vic Universitat Central de Catalunya), Avinguda Universitària 4-6, 08242 Manresa Barcelona, Spain; 3 Department of Rehabilitation Sciences and Physiotherapy, Faculty of Medicine and Rehabilitation Sciences, Ghent University, Sint-Pietersnieuwstraat 25, B-9000 Ghent, Belgium; 4 Department of Kinesiology, California State University, Northridge (CSUN), 18111 Nordhoff Street, 91330 Northridge, CA USA

**Keywords:** Electromyography, Fatigue, Muscle pattern, Low back pain, Semi-squat

## Abstract

**Background:**

Chronic low back pain (CLBP) has been associated with altered trunk muscle responses as well as increased muscle fatigability. CLBP patients and fatigued healthy subjects could experience similar neuromuscular strategies to attempt to protect the spine. The current study examined muscle activation differences between healthy and CLBP subjects following a perturbation. In addition, the possible role of muscle fatigue was evaluated by investigating the healthy control subjects in a non-fatigued and a fatigued condition. Both experiments were combined to evaluate possible similar strategies between CLBP and fatigued samples.

**Methods:**

Cross-sectional study where 24 CLBP subjects and 26 healthy subjects were evaluated. Both groups (CLBP vs. healthy) and both conditions (non-fatigued and a fatigued condition) were evaluated while a weight was suddenly dropped on a held tray. Erector spinae, multifidus, obliques and biceps brachii were recorded using surface electromyography. Variables describing the bursts timing and variables describing the amount of muscle activity (number of bursts and amplitude increase) post impact were studied. The analysis between groups and conditions was carried out using ANOVAs with repeated measurements for the muscle factor.

**Results:**

CLBP subjects reacted similarly to healthy subjects regarding muscle activity post impact. However, the CLBP group showed temporal characteristics of muscle activity that were in between the fatigued and non-fatigued healthy group. Clear differences in muscle activity were displayed for healthy subjects. Fatigued healthy subjects presented more reduced activity after impact (upper limb and trunk muscles) than non-fatigued healthy subjects and different temporal characteristic in the same way than CLBP patients. This same temporal characteristic with CLBP and healthy fatigued people was a delay of the first burst of muscle activity after impact.

**Conclusion:**

Though similar muscle pattern existed between CLBP and healthy people, CLBP temporal characteristics of muscle activity showed a pattern in between healthy people and fatigued healthy people. While the temporal muscle pattern dysfunction used by CLBP subjects could be related to maladaptive patterns, temporal and muscle activity characteristics used by healthy fatigued people may lead to back injuries.

## Background

Chronic low back pain (CLBP) is a multifactorial syndrome that represents a major problem throughout the world [1]. In recent years, trunk neuromuscular deficiency has been associated with low back pain: delays in activation (larger muscle activation latencies) [2, 3] and higher levels of muscle activation and trunk muscle co-contraction [3, 4]. These deficits have been related as a goal to protect from further pain, injury, or both, but in the same way as a possible source of further problems in the long term and have been suggested to contribute to CLBP [5]. For example, greater co-contraction strategies have been associated with the attempt to protect the spine, despite the possible overload of spine compression that results [3, 6]. Despite the strength of some of these hypotheses and findings, some studies show that the lack of differences in trunk muscles between CLBP and healthy subjects [7] could indicate that we do not fully understand all the variables that could influence the detection of dysfunctions.

Muscle activation levels and muscle activation latencies have been studied not only in back pain, but also in fatigue. When fatigued healthy individuals were exposed to a sudden perturbation, some studies demonstrated increases in the electromyographic (EMG) amplitude as a strategy to compensate for the loss of force production [8]; others demonstrated lower trunk muscle co-contraction compared to non-fatigued people, which was associated to spinal stability vulnerability [9]; and others found longer activation latencies as a deterioration of responsiveness and precision of the neuromuscular spindle system [10].

Most studies about muscle onset timing following an external perturbation in CLBP individuals or fatigued subjects were only performed by evaluating the latency or amplitude of the onset, without analysing the rest of the muscle responses. To know the whole muscle behaviour throughout the time could imply some important clinical considerations regarding treatment or prevention interventions in those populations. Moreover, to the authors’ knowledge, there are no studies in which measurements of muscle reactions following a perturbation are compared between CLBP patients and healthy controls and in which possible differences are compared with what happens when the healthy population is fatigued. CLBP patients and fatigued healthy subjects could experience similar neuromuscular strategies to attempt to protect the spine. Therefore, the current study had three objectives: a) to evaluate differences between healthy subjects and those suffering from CLBP in the sequence and amount of EMG muscle activity that occur after a perturbation during a functional position; b) to evaluate in an analogue way the effect of fatigue in healthy subjects and c) to evaluate similar compensatory strategies which might be used by both CLBP and fatigued subjects.

## Methods

### Subjects

Twenty-four subjects with CLBP and 26 healthy subjects were recruited (25–55 years old). The inclusion criteria for CLBP subjects were constant or nearly constant pain in the lower back for over a year with painful periods of at least 7 on the numeric rating scale (NRS) (segmented numeric version of 100-mm Visual Analog Scale with 0–10 integers). Those subjects who had other health problems that could affect recorded or outcome data were excluded. Those individuals who at the time of data collection were suffering pain with more than 4 on NRS were asked to come back on a later occasion. Subjects with CLBP were recruited from the Althaia Foundation (Spain) and healthy subjects were recruited from Manresa University (Spain) matched with CLBP subjects in age, gender, height and body mass (Table 1). Each subject signed an informed consent form. The project was approved by the local Ethics Committee.

**Table 1:** Group sample description and significance values of the t-test and chi square test

	H		CLBP		Sig (p)
N	26		24		
Age (years)	39.11	(8.73)	38.82	(8.16)	0.904
Gender^Ɨ^ (Male%)	34.6		37.5		0.628
Height (cm)	166.31	(8.51)	165.20	(8.45)	0.185
Body Mass (Kg)	63.83	(10.89)	66.52	(13.69)	0.443
Body Mass Index ^a^	22.96	(2.71)	24.30	(4.23)	0.194
Waist-hip ratio ^b^	0.78	(0.08)	0.81	(0.09)	0.248
Load during the test (Kg)	3.97	(1.29)	4.28	(1.34)	0.421

*H* healthy subjects, *CLBP* subjects with chronic low back pain; Values are mean (standard deviation) for continuous variables and n and % for categorical variables. Ɨ Chi-square for categorical variables

^a^ Data transformed using ln because normality test failed

^b^ Data transformed using 1/x because normality test failed

### Procedures

**Fig. 1:**
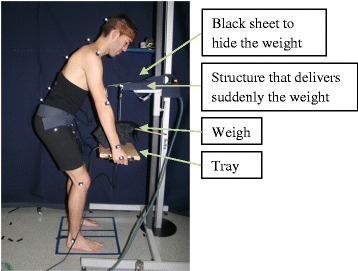
External perturbation test

**Fig. 2:**
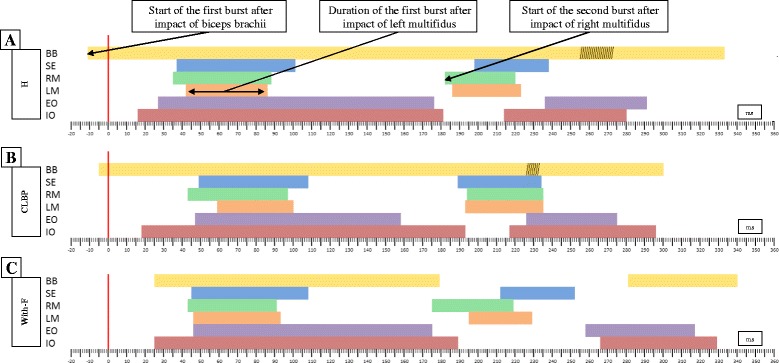
Temporal representation of the average value of the bursts of each muscle (biceps brachii (BB) thoracic spinal erector (SE) *right* multifidus (RM), *left* multifidus (LM), external oblique (EO), internal oblique (IO)) for both groups and for the condition of fatigue. **a** Group without lumbar pathology (H); **b** Group with nonspecific chronic low back pain (CLBP); **c** Condition of fatigue (With-F). The coloured bars represent, for different muscles, the moment when the first and second bursts after impact starts and their duration. The striped areas represent the parts of the bursts belonging to two different bursts (overlapping)

Three equal sessions on three different days were designed for each subject to collect the external perturbation test data (EPT) (Figs. 1 and 2). Anthropometric measures necessary for calculating the weight applied in the EPT were collected at the beginning of each testing session. Finally, six attempts of the EPT were carried out separated by a 30-s interval. Subjects were asked to stand in a bipedal semi-squat position holding a tray with both hands in front of the instrument that would release the load (Fig. 1). They had to stand in such a way that their acromion reached an elevation equal to 94% of individual stature. The 94% elevation was chosen using visual inspection to reproduce the semi-squat posture. At the sound of a buzzer, at a random interval of two to ten seconds, a weight was dropped on the tray without warning and without being seen, causing a sudden perturbation in the flexion direction. The load applied was a weight released from 15 cm above the tray and corresponded to 3.5% of the predicted maximum extensor moment (PMEM) of each individual. The trunk PMEM was calculated according to gender described previously [11]:

$$ \mathrm{Women}:\ \mathrm{PMEM} = 6.506 \ast \mathrm{FFBM}\ \hbox{-}\ 47.2 $$

$$ \mathrm{Men}:\ \mathrm{PMEM} = 9.227 \ast \mathrm{FFBM}\ \hbox{-}\ 172.9 $$

whereby FFBM is the fat-free body mass estimated according to the method of Durnin and Womersley [12].

$$ \mathrm{Density} = \mathrm{c}\hbox{-} \mathrm{m} \ast \log\ \mathrm{skinfold}\ \left(\mathrm{triceps} + \mathrm{biceps} + \mathrm{subscapular} + \mathrm{supra}\hbox{-} \mathrm{iliac}\right)\ \%\ \mathrm{fat}=\left(4.95/\mathrm{density}\ \hbox{-}\ 4.50\right)*\ 100 $$

$$ \mathrm{FFBM} = \mathrm{weight} \ast \left(100\ \hbox{-}\ \%\mathrm{fat}\right)/100\Big) $$

A fatigue protocol was added only in the second session and only for the healthy group after the sixth EPT. This consisted of maintaining a weight corresponding to 40% of the PMEM in the same position as long as they could. After the fatigue protocol, another six EPT with 30–40 s delay between fatigue and EPT attempts was performed. The healthy group without fatigue was considered as condition Non-F and the same group after the fatigue protocol was considered as condition With-F.

### EMG analysis and data processing

A ME6000 electromyography system (Mega Electronics, Kuopio, Finland) was used to register the EMG signals. EMG recordings were conducted during all the MVC efforts and for all the EPT attempts. Right thoracic spinal erector (SE), right multifidus (MR), left multifidus (LM), right biceps brachii (BB), right external oblique (EO) and right internal oblique (IO) were recorded. Adhesive surface electrodes (Ambu-Blue-Sensor, M-00-S, Denmark), were placed 2-cm apart according to anatomical recommendation of the SENIAM [13] except for IO [14]. The skin was prepared according to SENIAM specifications [13]. The EMG data were collected at 2000 Hz and were amplified with a gain of 1000 using an analogue differential amplifier and a common mode rejection ratio of 110 dB. The input impedance was 10 GΩ. A Butterworth band pass filter of 8–500 Hz (-3 dB points) was used.

An accelerometer (measuring range 10G) located at the bottom of the tray and synchronised with EMG was the indicator of the weight drop. The algorithm used was based on averages and standard deviations of the EMG record baseline amplitude, designed and validated by our group. A validated algorithm [15], determining the points where the amplitude of the EMG record increases compared to the baseline record and decreases to return to the same baseline, was used to identify EMG bursts. The bursts that were separated by a period of less than 15 ms were considered as a single burst and bursts lasting less than 15 ms were not taken into account.

Two categories of variables were analysed: 1) variables describing the timing including start and duration of the first and second burst and duration of co-contraction between the main trunk muscles after the impact. The duration of co-contraction was considered as the milliseconds during SE-OE, SE-IO or EO-IO generated a burst in the same period; and 2) variables describing the amount of muscle activity post impact. The amount of activity was analysed based on the number of bursts after the impact and the amplitude increase after the impact (ratio of root mean square (RMS) of the post impact EMG signal amplitude and RMS of the pre-impact EMG signal amplitude). The RMS was determined during the interval 500 milliseconds before and after impact. For all variables, the median of all attempts (18 attempts for each individual in the comparison between CLBP vs. healthy and 6 attempts for the condition With-F vs. Non-F) was calculated as a representative value of each individual [16].

### Statistical analysis

Demographic differences between groups were studied using independent t-tests for parametric variables and a chi-square for non-parametric variables. The analysis between groups (CLBP vs. healthy) was carried out for each variable using mixed group by muscle ANOVAs with repeated measurements for the muscle factor [16, 17]. Post hoc Tukey corrections were performed to analyse the muscle factor significance. Simple factor analysis was used to further analyse a significant group by muscle interaction, and finally post hoc Bonferroni corrections were performed to analyse the significance between muscles when the group was fixed.

For the analysis between conditions (With-F vs. Non-F) the same statistical procedures were used for the analysis between groups, with the exception that the condition factor (Non-F, With-F) was treated in all cases as repeated measurements. In all the variance analyses the value of *eta partial square* was considered as an estimate of the size of the effect. In the results section, all significant values (<0.05) have been reported.

## Results

The CLBP and healthy groups showed no differences in their baseline demographic and anthropometric characteristics. Similar weights were applied during the test between groups (Table 1). The specific pain history (intensity and duration) of the CLBP subjects during the previous year to data collection can be found in Table 2. As a summary of the variables describing the timing, Fig. 2a, b and c show graphically the average of these variables for the CLBP, healthy group and fatigue condition.

**Table 2:** Pain history, intensity and duration, of the group with CLBP

	Mean	SD	Maximum	Minimum
Maximum level of low back pain in the past year	8.81	1.13	10	7
Number of days without pain in the past year	37.04	51.82	174	0
Number of days with low pain in the past year	149.37	86.12	303	0
Number of days with moderate pain in the past year	119.29	51.94	210	22
Number of days with high pain in the past year	57.04	68.72	262	0

The level of pain was analysed using the numeric rating scale (NRS) for pain (scale from 0 to 10). Low pain was considered a pain that ranged between 1 and 3.9 on the NRS scale, medium pain was considered to be between 4 and 7.9 on the NRS scale, and high pain was considered to be between 8 and 10 on the NRS scale

**Fig. 3:**
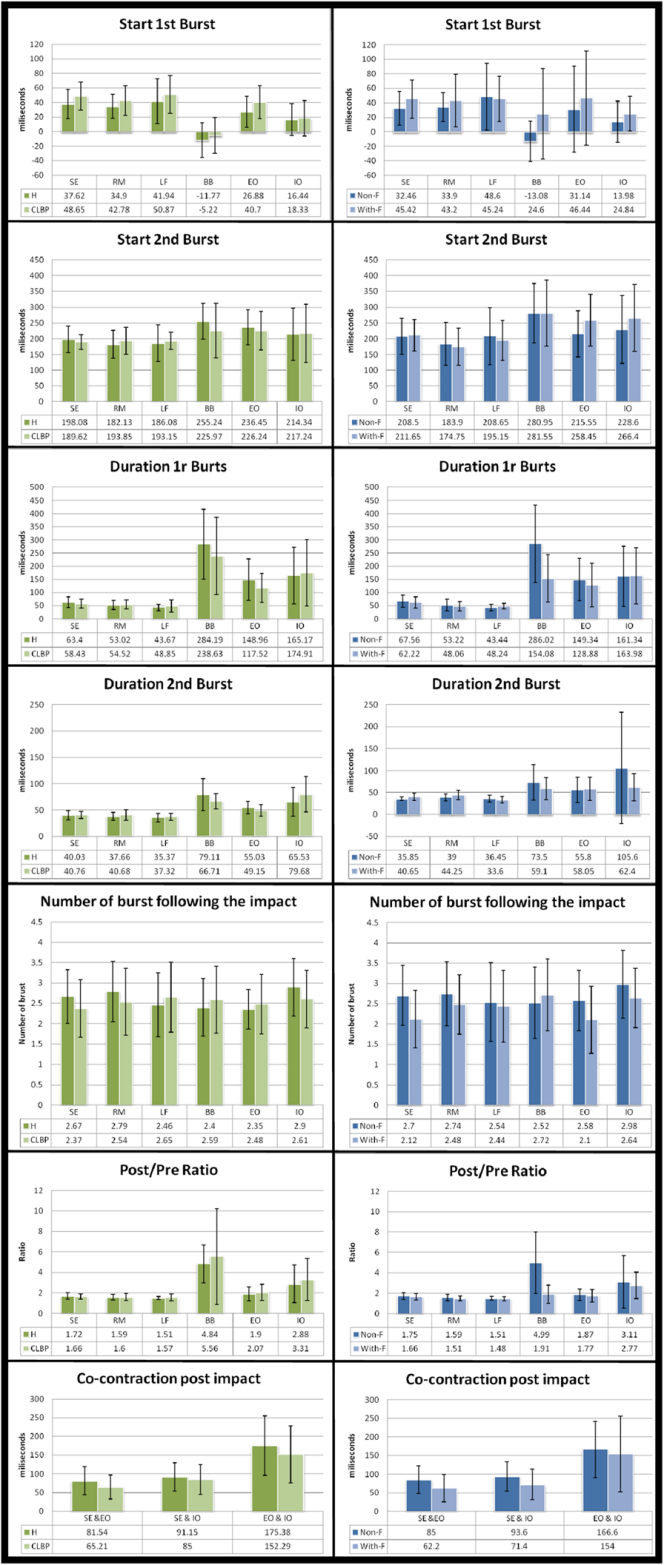
Mean and standard deviation (error bars) of each muscle (biceps brachii (BB), thoracic spinal erector (SE), *right* multifidus (RM), *left* multifidus (LM), external oblique (EO), internal oblique (IO)) of variables describing the timing and variables describing the amount of muscle activity post impact between healthy subjects and those with chronic low back pain and between healthy subjects without fatigue and healthy subjects with fatigue

Regarding differences between groups (CLBP vs healthy), statistical analysis of the variables describing the timing and the quantitative variables of muscle activity (number of bursts following the impact and the increase of activity after the impact) showed no differences between CLBP and healthy subjects (Table 3 and Fig. 3).

**Table 3:** Comparison between healthy and chronic low back pain subjects

	Principal effect	gl	F	Sig (p)	pŋ2	Power	Post hoc/Simple factor
Start 1st *Burst*	Group	47(1)	3.834	0.056	0.075	0.483	
	Muscle		58.069	≤0.001	0.553	1.000	BB < IO < SE,RM,LM,EO
	Interaction		0.555	0.686	0.012	0.179	
	Group mean difference (95% CI) = 8% (−17, 0.0) for start 1rst *Burst*						
Start 2nd *Burst*	Group	34(1)	0.270	0.607	0.008	0.080	
	Muscle		5.250	0.002	0.134	0.933	BB,EO > SE,RM,LM
	Interaction		0.565	0.651	0.016	0.168	
	Group mean difference (95% CI) = 4% (−13, 22) for start 2nd *Burst*						
Duration 1st *Burst*	Group	47(1)	1.038	0.314	0.022	0.170	
	Muscle		58.160	≤0.001	0.553	1.000	BB > EO,IO > SE > LM
	Interaction		1.009	0.378	0.021	0.240	RM < BB > EO,IO
	Group mean difference (95% CI) = 11% (−11, 33) for duration 1st *Burst*						
Duration 2nd *Burst*	Group	34(1)	0.010	0.919	0.000	0.051	
	Muscle		33.709	≤0.001	0.498	1.000	BB,IO > EO > SE,RM,LM
	Interaction		2.408	0.089	0.066	0.508	
	Group mean difference (95% CI) = 0% (−6, 5) for duration 2nd *Burst*						
Num *Bursts* post impact	Group	47(1)	0.271	0.605	0.006	0.080	
	Muscle		1.596	0.181	0.033	0.469	
	Interaction		1.596	0.181	0.033	0.469	
	Group mean difference (95% CI) = 0.56% (−0.16, 0.27) for Num *Bursts* post impact						
Post/Pre Ratio	Group	47(1)	0.976	0.328	0.020	0.162	
	Muscle		39.166	0.000	0.455	1.000	BB > IO > EO > RM,LM LM < SE < BB > IO
	Interaction		0.425	0.618	0.009	0.111	
	Group mean difference (95% CI) = 0.22% (−0.67, 0.22) for Post/Pre Ratio						
Co-co post impact	Group	48(1)	1.587	0.214	0.032	0.235	
	Muscle		71.833	≤0.001	0.599	1.000	SE&EO < SE&IO < EO&IO
	Interaction		0.554	0.499	0.011	0.120	
	Group mean difference (95% CI)= 15.19% (-9.05, 39.43) for co-co post impact						

Significant ANOVA results corresponding to the comparisons between healthy subjects and those with chronic low back pain (group factor) and between muscles (muscle factor) for variables describing the timing and variables describing the amount of muscle activity post impact. *Num bursts post impact* number of bursts following the impact, *Post/Pre Ratio* ratio of root mean square of the post impact EMG signal amplitude and pre impact EMG signal amplitude, *Co-co post impact* co-contraction levels after the impact, *BB* biceps brachii, *SE* thoracic spinal erector, *RM* right multifidus, *LM* left multifidus, *EO* external oblique, *IO* internal oblique

Regarding differences between conditions (With-F vs Non-F), statistical analysis of the variables describing the timing showed a significant delay of the first burst for the With-F condition when compared to the Non-F condition (Table 4 and Fig. 3). Similarly, the With-F condition showed a significantly shorter length of the BB first burst when compared to the Non-F condition (Table 4 and Fig. 3). On the other hand, the co-contraction levels showed significant differences between the two conditions with lower values in the With-F compared to the Non-F condition (Table 4 and Fig. 3). In the analysis of the quantitative variables, the condition With-F compared to Non-F showed a smaller number of bursts after impact and lower values of increase of activity after the impact; although the presence of significant interaction (*P* < 0.001) showed that lower values of increase of activity after the impact in the With-F condition was only for the SE and BB muscles (Table 4 and Fig. 3).

**Table 4:** Comparison between non-fatigued healthy subjects and fatigued healthy subjects

	Principal effect	gl	F	Sig (p)	pŋ2	Power	Post hoc/Simple factor
Start 1st *Burst*	Condition	24(1)	4.739	0.040	0.165	0.552	With-F > Non-F
	Muscle		9.279	≤0.001	0.279	0.996	BB < SE,RM; IO < LM; BB < EO
	Interaction		2.234	0.093	0.085	0.540	
	Group mean difference (95% CI) = 14% (−27, 1) for start 1st *Burst*						
Start 2nd *Burst*	Condition	9 (1)	0.693	0.427	0.071	0.116	
	Muscle		3.228	0.057	0.264	0.571	
	Interaction		0.623	0.568	0.065	0.146	
	Group mean difference (95% CI) = 10% (−38, 18) for start 2nd *Burst*						
Duration 1st *Burst*	Condition	24(1)	15.726	0.001	0.396	0.967	With-F < Non-F
	Muscle		42.055	≤0.001	0.637	1.000	BB > EO > SE > RM,LM; IO > SE > RM,LM
	Interaction		8.794	0.001	0.268	0.963	BB = With-F < Non-F
	Group mean difference (95% CI) = 26% (−12, 39) for duration 1st *Burst*						
Duration 2nd *Burst*	Condition	9(1)	1.214	0.299	0.119	0.167	
	Muscle		3.023	0.098	0.251	0.413	
	Interaction		1.570	0.242	0.149	0.240	
	Group mean difference (95% CI) = 8% (−8, 24) for duration 2nd *Burst*						
Num *Bursts* post impact	Condition	24(1)	7.679	0.011	0.242	0.758	With-F < Non-F
	Muscle		1.574	0.198	0.062	0.422	
	Interaction		2.509	0.053	0.095	0.661	
	Group mean difference (95% CI) = 0.26% (−0.06, 0.45) for num *Bursts* post impact						
Post/Pre Ratio	Condition	24(1)	28.323	0.000	0.541	0.999	With-F < Non-F
	Muscle		20.182	0.000	0.457	1.000	BB,IO > EO > LM; BB,IO > SE > RM,LM
	Interaction		13.798	0.000	0.365	0.992	SE = With-F < Non-F
							BB = With-F < Non-F
	Group mean difference (95% CI) = 0.61% (−0.37, 0.85) for start post/Pre Ratio						
Co-co post impact	Condition	24(1)	4.625	0.042	0.162	0.542	With-F < Non-F
	Muscle		33.856	≤0.001	0.585	1.000	SE&EO,SE&IO < EO&IO
	Interaction		0.399	0.577	0.016	0.098	
	Group mean difference (95% CI) = 19.20% (−0.77, 37.62) for co-co post impact						

Significant ANOVA results corresponding to the comparisons between healthy subjects without fatigue and healthy subjects with fatigue (group factor) and between muscles (muscle factor) for variables describing the timing and variables describing the amount of muscle activity post impact. *Num bursts post impact* number of bursts following the impact, *Post/Pre Ratio* ratio of root mean square of the post impact EMG signal amplitude and pre impact EMG signal amplitude, *Co-co post impact* co-contraction levels after the impact, *BB* biceps brachii, *SE* thoracic spinal erector, *RM* right multifidus, *LM* left multifidus, *EO* external oblique, *IO* internal oblique

**Fig 4:**
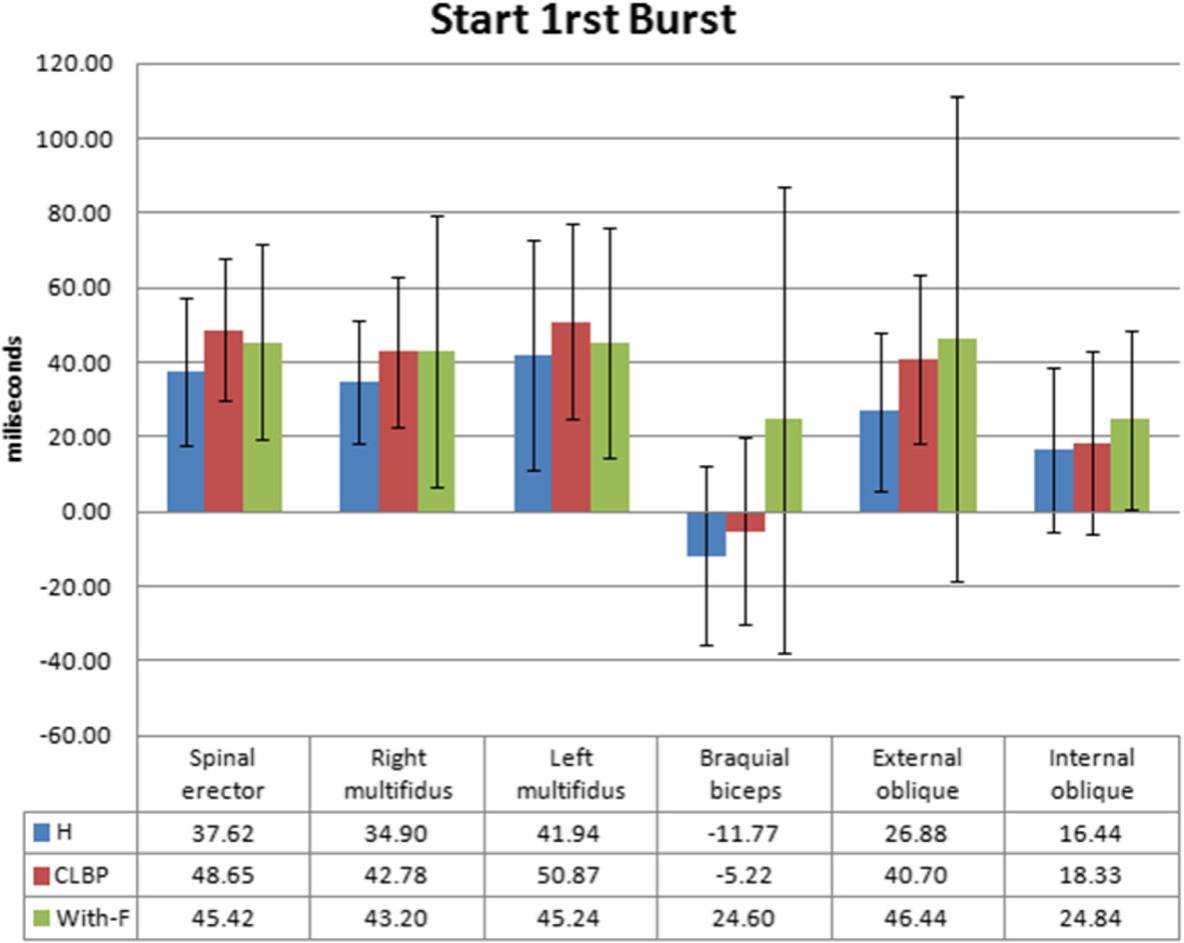
Mean and standard deviation (error bars) of each muscle (biceps brachii (BB), thoracic spinal erector (SE), *right* multifidus (RM), *left* multifidus (LM), external oblique (EO), internal oblique (IO)) of the variable of first burst post impact between healthy subjects, those with chronic low back pain and between healthy subjects with fatigue

Although not statistically significant, the CLBP showed a pattern of response for first burst delay closer to fatigued than non-fatigued healthy subjects (Fig. 4)

.

## Discussion

The present study shows that people with CLBP used similar muscle pattern compared to the healthy group when reacting to an unexpected external load. On the other hand, fatigue caused muscle pattern changes regarding temporal and amount of muscle activity characteristics that were different when compared to those of the non-fatigued group. Although not statistically significant, the CLBP showed a pattern of response for some temporal variables closer to fatigued than non-fatigues healthy subjects (Fig. 4). This similarity may indicate that some behavioural characteristics could be shared between CLBP and healthy fatigued groups. Other studies have also shown similar characteristics when comparing these two groups, e.g. proprioception alteration [18, 19]. The fact that the common temporal changes are less obvious in subjects with CLBP could be caused by the greater heterogeneity in characteristics presented by this group. However, it should be taken into account that the muscular alteration in individuals with CLBP is maintained over time, unlike the With-F group who only has this condition on a temporary basis.

Greater latency in the CLBP group in the activation of the first burst for most muscles has been determined by most authors [2, 3, 20]. However, in this study, no significant differences between CLBP and healthy groups were found in the first muscular activation delay after an external perturbation. Although not statistically significant, the CLBP showed a pattern of response of the first burst latency closer to fatigued than non-fatigued healthy subjects, with the fatigued group showing a clear delay in the first burst activation (Fig. 4). This tendency could support current theories [16, 21, 22] which describe delays in muscle activation as a phenomenon that decreases the control of the spine possibly leading to chronic pain. The lack of findings in this study and other authors concerning this issue [7] could be explained by different factors. The small sample size and large variability among CLBP subjects [5] could contribute to diminished power. Future studies with a larger sample size and/or sub-classifications of CLBP would be required to clarify this issue. In addition, the specific characteristics of the test used in the different studies could also contribute to limited detection of greater latencies in CLBP as described by others [2, 3, 20]. Controlling the pre-activation trunk muscles and using a more fixed position [7] could imply an experimental condition where CLBP muscle deficits were not observed. In healthy people, abdominal and trunk muscle pre-activation had showed increases in spinal stiffness and stability [6]. Stable contexts could not be those situations where CLBP subjects present deficits in spine control. Moreover, one may interpret that the more real the position of the test (this study vs. others [7]), the more it relates to CLBP subjects’ daily lives. The semi-squat position, used in the current study, is a recommended posture to handle physical efforts made at the spine level, and it is frequently used without external stabilization. Also, unexpected perturbations could be experienced in daily activities. Less ability to protect the spine by CLBP in this frequent situation seems more relevant than results from an unusual context.

Subsequent muscular reactions (EMG bursts) also seem to be important to guarantee spinal protection. In the current study, the CLBP group showed no differences when compared to the healthy group without fatigue. The current results can only be compared with other studies investigating the first reaction response since, to the authors’ knowledge, other research up to now, does not consider subsequent reactions. Only one study evaluated the completion time of the first burst without observing differences between the CLBP group and the healthy group in that parameter [17]. The variability among subjects in the motor recruitment pattern [5] could be greater in subsequent muscular reactions rather than in the first reaction. This could be the reason for the lack of significance among the subsequent muscle reactions found in this study or other ones.

CLBP showed similar amount of activity than healthy group after the impact as well as similar co-contraction based on the calculation of the burst synchronisation after impact in the three muscle groups. However, in different tasks, other studies found in CLBP people more muscle activation in certain muscles [7] as well as increased agonist and antagonist activity attributing to increased muscle co-contraction [4]. Both variables of the current study that describe the amount of muscle activity post impact (burst number and amplitude increase) were relative of pre-impact activity without considering the possible absolute group differences on the EMG normalized amplitude. That could be a reason explaining why other studies show discrepancies regarding muscular activity in CLBP subjects [4, 7]. Interpreting together those results of the amount of muscle activity, one may conclude that CLBP subjects may be using more activity than healthy ones but not a large increase of muscular activity after the external perturbation. Regarding the co-contraction parameter, this study cannot be compared directly with those studies where co-contraction was calculated as an amplitude increment of agonist and antagonist muscles [4]. The co-contraction parameter of this study is based on muscle onset and offset times so similar co-contraction between groups is because no more burst synchronisation is found in CLBP group compared with the healthy one. The co-contraction parameter of this study may be contrasted with studies like Mehta et al. [17], Radebold [3] and Cholewicki [23]. Mehta et al [17] analysed the coincidence in time of the first burst on a sudden perturbation showing a lower synchronisation in CLBP subjects. Conversely, Radebold [3] and Cholewicki [23] determined that greater co-contraction occurred in the CLBP group, as they observed less muscleagonist deactivation once the load was withdrawn when compared to the healthy group. Considering all CLBP evidence together, one might conclude that the increase of muscle activity and the presence of co-contraction are strategies used by people with CLBP to reduce pain [5], but are not present in all types of tasks. It appears that in those tasks that require a sudden increase in muscle activity to control the spine (this study among others [17]), the delay in muscle activation could make synchronisation impossible which undermines the possibility of co-contraction. Conversely, in slower tasks [4] or in tasks with an initial considerable co-contraction [3, 23], increased slowness in muscular reaction would not prevent the co-contraction strategy. Similarly, even though absolute EMG normalized amplitude differences between groups are not assessed in this study, muscle activity increase as a strategy by CLBP subjects to reduce pain [5] could be more difficult presented after sudden perturbation than in a static position [24] or when undertaking a slower task [4]. A recent new theory regarding adaptation to pain supports this task dependency interpretation [5]. In sudden perturbation, delays in muscle activation and the difficulty of using previous described strategies to control the spine [3, 6] could imply a vulnerability for CLBP subjects and may play a role in the chronification process. Muscle training to improve muscle coordination and the quickness of muscle responses could be a strategy to improve CLBP dysfunctions. Moreover, one might consider the need for functional exercises as a treatment of CLBP (semi-squat among others).

Regarding the fatigue condition, greater latencies in the activation of the first burst, and alteration in subsequent reaction times existed (earlier times in the deactivation of the first burst of the BB muscle and less co-contraction of SE, EO and IO). Healthy subjects seem to show in the fatigue condition a similar phenomenon that decreases the control of the spine as that described for temporal alteration in CLBP [16, 21, 22]. In this transitory situation of fatigue, the lack of control seems to be much more pronounced than in CLBP subjects and could lead to a tissue injury. Some authors found similar results to the current study regarding the first onset [10], while others have not observed latency differences in the first burst [8, 25]. Again, literature discrepancies could be explained by the specific characteristics of the perturbation, different fatigue levels achieved prior to the reaction test and the task used to induce fatigue [26]. Future studies analysing the same subjects with different tasks and methodologies could help to resolve this issue. Contrary to the CLBP group, the fatigue condition showed signs of reduced activity after the impact compared with non-fatigue condition (smaller amount of bursts and smaller increase in muscle activation after impact of some muscles). This reduction may indicate that fatigue leads to a lesser control of the spine overloading on different structures and is more likely to result in back injury. Some considerations must be taken against fatigue to prevent spine overloading.

## Limitations

CLBP subjects constitute a very heterogeneous and multifactorial group. Even though the sample size in the present study could be larger, we have used a similar or larger sample size than those currently used in similar research studies [8, 16]. For this reason results should be interpreted cautionarily and considered as exploratory. Moreover, we must assume that the current experimental findings could be extrapolated only to people with similar demographic and anthropometric characteristics (age, body mass, etc.). It should be noted that this study has evaluated a static and very specific task that is not representative of the multiple dynamic tasks that are carried out by the individuals in their daily lives. Another limitation is the absence of the abdominal transverse or other task-contributor muscles, which may mean that we have overlooked muscles with a role in the muscle recruitment patterns and that could present different behaviour between groups (CLBP vs. healthy) or between conditions (With-F vs. Non-F). Moreover, the fact that we did not conduct a complete bilateral assessment could mask some dysfunctional muscle behaviours related to CLBP or With-F groups.

Finally, two important aspects must be considered in the interpretation of temporal electromyographic data: a) the difficulty determining the bursts’ onset. Despite the use of an algorithm designed specifically for this purpose, greater initial muscle activity can lead to greater variability in the determination of the onset and b) it must be noted that the actual muscle contraction itself is not being recorded, as the EMG signal represents the muscle’s electrical activity. The type of muscle fibres, the electrode’s distance to the innervation zone centre, for example, may entail certain differences between the electrical activity records of the different muscles and the delay that exists in the final contraction [27].

## Conclusion

When controlling the trunk after an unexpected external perturbation, CLBP subjects seemed to react similarly to healthy subjects regarding muscle activity post impact. However, the CLBP group showed temporal characteristics of muscle activity that were in between the non-fatigued and fatigued healthy groups. Clear differences in muscle activity were displayed by the healthy subjects in the same situation. Fatigued subjects used different muscle patterns when compared to healthy subjects without fatigue. They reacted with greater muscle latencies in the activation of the first burst among some other temporal characteristics. In addition, they presented more reduced muscle activity after impact than healthy subjects. A temporal characteristic between CLBP and healthy fatigued people was a delay of the first burst of muscle activity after impact. We suggest that these muscle patterns present in CLBP and healthy fatigued subjects and especially in sudden perturbations could imply a vulnerability and may play a role in CLBP dysfunction or may lead to back injuries in fatigued people.

## Abbreviations

BB: Right biceps brachii
CLBP: Chronic low back pain
EMG: Electromyographic
EO: Right external oblique
EPT: External perturbation test data
FFBM: Fat-free body mass
IO: Right internal oblique
LM: Left multifidus
MR: Right multifidus
Non-F: The healthy group without fatigue
NRS: Numeric rating scale
PMEM: Predicted maximum extensor moment
RMS: Root mean square
SE: Right thoracic spinal erector
With-F: The healthy group after the fatigue protocol
